# Sustainable by Design: Digital Health Business Models for Equitable Global Health Impact in Low-Income and Low-Middle-Income Countries

**DOI:** 10.1016/j.mcpdig.2025.100261

**Published:** 2025-09-04

**Authors:** Elvin Irihamye, Justin Hadad, Natasha Ali, Bruno Holthof, Francis Wafula, Chris Paton, Mike English, Shobhana Nagraj

**Affiliations:** a NDM Centre for Global Health Research, Nuffield Department of Medicine, University of Oxford, United Kingdom; b Department of Economics, University of Oxford, United Kingdom; c Institute of Healthcare Management, Strathmore University, Nairobi, Kenya; d The Liggins Institute, The University of Auckland, New Zealand; e KEMRI-Wellcome Trust Research Programme, Nairobi, Kenya; f Department of Public Health & Primary Care, University of Cambridge, United Kingdom; g East London NHS Foundation Trust, United Kingdom

## Abstract

This study explores challenges and potential strategies related to sustaining digital health business models and markets in low-income and low-middle-income countries using a critical interpretive synthesis approach. We extracted 21 articles from a database search that yielded over 1300 hits and used insights from 7 expert reviewers with experience operating or funding digital health companies in low-middle –income countries. Findings reveal 4 key challenges: (1) internal challenges related to managing value creation for complex stakeholder networks and external challenges related to (2) infrastructure, (3) financing, and (4) regulation. Entrepreneurs must address these through iterative business strategies, but broader market-shaping interventions remain essential. Such interventions could include facilitating strategic partnerships, fit-for-purpose regulation, enhancing public procurement, and innovative financing instruments. Health systems can tailor interventions around their unique contexts by prioritizing technologies, recruiting local market participants, analyzing shared barriers in the business environment, focusing on feasible interventions, and iterating to sustain a competitive environment.

**Article Highlights**

• Many low-income and low-middle-income country digital health initiatives struggle to move beyond pilots into sustainable, scaled products with system-wide impact.
• Companies face internal hurdles such as identifying feasible opportunities, measuring and communicating value, and expanding withing existing systems and workflows.
• External barriers are common, including infrastructure challenges, unclear or a lack of regulation, and limited or unreliable financing.
• Digital health initiatives will need to embrace iterative or agile strategies to cost-effectively scale their products and services.
• Market-shaping by governments and nongovernmental organizations may help to align private sector incentive to public health goals through improved regulation, public procurement, innovative funding, and partnership support.

Globally, health systems face complex challenges delivering high-quality, equitable, and cost-effective health care. To improve care delivery with limited resources, low-income and low-middle-income countries (LMICs) are increasingly adopting digital tools to capture health care data, generate analytics, and support innovative clinical and operational processes.^1^^,^^2^ Examples of promising digital health intiative (DHIs) include electronic health records, teleconsultations, artificial intelligence–based virtual assistants, and clinical decision intelligence systems.^3^

Yet, despite significant investments and techno-optimism from global health funders and purchasers, DHIs have largely been limited to discrete short-term activities like collecting data and reporting, with most initiatives failing to achieve scale and contribute to health system transformation.^2^^,^^4^^,^^5^ Market failures, or the lack of a sustainable purchasing landscape for innovations tailored to public health challenges, are one of the key reasons for this lack of scale and sustainability.^2^^,^^6^^,^^7^ Digital health companies serving public health systems even in high-income countries face immense challenges remaining financially viable beyond a pilot stage. Moreover, organizations in LMICs face additional infrastructural, financial, and regulatory barriers.^5^^,^^8^, ^9^, ^10^, ^11^ However, potential solutions to create a more enabling market may exist.

Similar market failures in the past have historically limited the reach of many pharmaceuticals and other essential medicines in LMICs. In response to these challenges, governments, donors and other groups have strategically deployed bespoke financial agreements, strategic partnerships, and other means to incentivize private sector production and distribution of medications in LMICs.^12^, ^13^, ^14^, ^15^ Such interventions have resulted in the expansion of access to essential medicines for millions of patients in LMICs by aligning the business models of the private sector to public health needs.^12^, ^13^, ^14^, ^15^

The success of market-based strategies in expanding access to pharmaceuticals raises the question of whether such interventions could help expand access to private sector DHIs, which tend to be less disease focused, more longitudinal, and more iterative in design and deployment in comparison with pharmaceutical interventions.^16^^,^^17^ Thus, a clearer understanding of the challenges to business model sustainability faced by DHI companies in LMICs might inform how governments and global health funders could adapt market-shaping tools at local, regional, and international levels to incentivize DHI businesses to more effectively tackle their health system challenges.

## Research Questions and AIMS

Sustaining and scaling private sector DHIs for global health impact remains a challenge in LMICs. To explore this, our study uses 2 interlocking approaches: deployment of critical interpretive synthesis (CIS) to characterize the challenges and use of a team of expert reviewers to help define an appropriate model.

Critical interpretive synthesis^18^ is an evidence synthesis method that uses interpretative qualitative analysis of literature to improve the understanding of phenomena. We used CIS to answer the following 3 key questions:

1) What are the major challenges to sustainability of DHI business models in LMICs?
2) Which internal business strategies could LMIC entrepreneurs employ to overcome the challenges?
3) What external market-shaping strategies could LMIC governments and nongovernmental organizations implement to support sustained development and scaling-up of DHIs for better performance of public health systems?

Second, we collected feedback from a panel of experts with experience in commercializing, funding, or consulting for digital health companies across the globe. Their input helped to assess the relevance of findings across different technologies and health system contexts, thus helping to refine the proposed market-shaping process to balance broad applicability across LMICs with an acknowledgment of the need for local adaptation.

## Methods

### Study Design

The CIS approach allowed us to investigate challenges and strategies for developing sustainable business models and markets for DHIs in LMICs. The CIS is well-suited for theoretical development within complex, interdisciplinary research areas. This is because it uses an inductive approach to analyzing purposively selected literature, with iterative interpretation and refinement of concepts and research questions as new insights emerge.^18^ The CIS entails an iterative multistep process that includes formulation of a research question, literature search, sampling, quality determination, data extraction, and creation of critical synthesizing arguments.^18^

### Search Strategy and Selection Criteria

A database search was conducted using 28 terms focused on business models and digital health (Supplemental Table 3, available online at https://www.mcpdigitalhealth.org/). The search was limited to English articles published between 2014 and May 14, 2024, and identified 893 relevant titles and abstracts, of which 21 full-text articles were included in the final analysis. Inclusion and exclusion criteria were applied during abstract and full-text screening to screen for relevance and quality. The title and abstract and full-text screening was conducted by a primary reviewer (E.I.). A second reviewer (N.A.) conducted a quality check on a random sample of the article count (20% of the total), and results were cross compared to improve the clarity of the inclusion/exclusion criteria and to add methodological rigor. Table 1 illustrates the inclusion and exclusion criteria for each stage of screening.

**TABLE 1:** Inclusion and Exclusion Criteria

Inclusion criteria	Exclusion criteria
Abstract screening	
Articles at the abstract screening phase were required to answer 1 of the following 2 key research questions for inclusion: 1. Does the article explicitly discuss the attributes or components of a “business model” in digital health? 2. Does the article implicitly or explicitly discuss challenges or strategies related to initiating or operating business models in digital health for financial sustainability or scalability?	Articles that are explicitly focused on only non-LMIC digital health companies or initiatives
Full-text screening	
Provide explicit appraisal or interpretation of a digital health business model, specifically addressing its construction or adaptation for sustainability and/or scalability. Case studies must include at least 1 explicit evaluation of a digital health company or project operating in an LMIC context.	1. Focused explicitly on digital health initiatives and case studies from high-income contexts 2. Lacked explanatory or interpretive power, often those that simply explained early-stage digital health company’s initial activities or motivations without any appraisal regarding their construction for financial sustainability or scalability. 3. Did not meet a minimal threshold of methodological quality.

LMIC, low-income and middle-income country.

### Determining Quality

**Figure 1:**
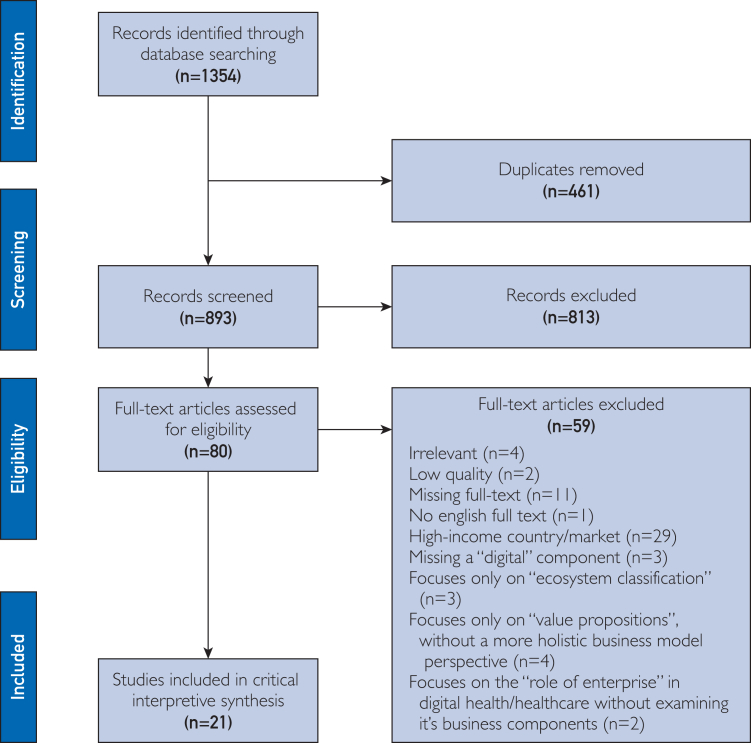
This PRISMA diagram shows the systematic process that was followed to include articles captured by our search terms.

Given the inherent reflexivity of CIS methodology and the absence of a strict hierarchy of study designs in qualitative research, we prioritized articles based on relevance to the research phenomena rather than applying inclusion criteria centered on rigid methodological standards. Thus, we applied a low threshold to quality appraisal, only screening out articles during the CIS that could be deemed fatally flawed for their low quality according to 5 key appraisal prompts (Supplemental Table 4, available online at https://www.mcpdigitalhealth.org/).^18^ Only 2 articles were excluded during full-text screening owing to low methodological quality. Figure 1 displays the PRISMA diagram for included studies.

### Data Extraction

The evidence extraction process involved 3 iterative steps supported by the authors: open coding, axial coding, and selective coding. First, open coding of an initial set of articles informed the creation of a structured data extraction form by focusing on identifying a unified conceptualization of a digital health business model from the digital health literature.^19^, ^20^, ^21^, ^22^, ^23^, ^24^, ^25^, ^26^, ^27^, ^28^, ^29^ The final conceptualization combined the overarching structure of the CompBizMod model (an evaluated business model framework for digital health), with the key subcomponents of Dr Alexander Osterwalder’s Business Model Ontology (a predominant business model ontology).^25^^,^^30^

#### Open Coding

This hybrid framework conceptualizes digital health business models as a dynamic, 4-component process of identifying value propositions,^11^^,^^19^^,^^20^^,^^24^^,^^25^^,^^27^^,^^29^^,^^31^, ^32^, ^33^, ^34^ creating or co-creating value,^11^^,^^19^^,^^24^^,^^25^^,^^27^^,^^29^^,^^31^^,^^33^ delivering and communicating value,^24^^,^^25^^,^^29^^,^^32^^,^^33^ and lastly, capturing value.^24^^,^^25^^,^^27^^,^^29^^,^^33^ These 4 components illustrated alongside with their associated subcomponents in Table 2, provided a unified analytical lens for examining digital health business models across heterogenous article types, which focus on a diverse array of technologies, market structures and health system contexts for implementation.

**Table 2:** Components and Subcomponents of a Business Model in Digital Health

Key business model components	Key subcomponents
Value proposition: The unique benefit or solution a product or service offers to meet the needs of 1 or more stakeholders in the health system.	Customer segments: The groups of people or organizations that your business can service. Customer value proposition: The core offering (product, service, and feature) that solves a problem or fulfills a need for a specific customer segment.
Value creation: The process of creating products or running services that improve the quality, access, cost, or operational efficiency of care delivery.	Key activities: The essential actions a business performs to deliver the value proposition. Key resources: The key assets (eg, people, funding, and technology) required to create and offer the product or service. Key partners: External implementers, organizations, and stakeholders who help reduce risks or optimize operations
Value communication and delivery: The approach to both disseminating the product or service and demonstrating its worth.	Channels: The methods (eg, physical or digital) through which a value proposition is communicated and delivered to customers and key partners. Customer relationships: The type of relationship established with your Customers and/or key partners to improve satisfaction and retention
Value capture: The process of retaining a portion of the value created through profit or another strategic benefit necessary for the scalability of the company.	Revenue streams: The ways in which a company generates income from delivering value to each of its key customer segments. Cost structure: The costs a company incurs in creating and delivering the value proposition. These costs can be either variable, an expense proportional to how much a company sells or produces, or fixed.

However, given variation in the level of descriptive and longitudinal evidence encountered during open coding, it became necessary within the extraction form to distinguish between two key dimensions of business model evidence: (1) evidence pertaining to the initial design and selection of business models, capturing the range of strategic decisions available or made at the outset, and (2) evidence related to how these models evolved and were sustained (or not sustained) over time, focusing on the ongoing management and adaptation of prior decisions in response to real-world challenges and opportunities. Thus, short quotes related to the 4 business model components were extracted from the 21 articles and further stratified into either the first or second categories.

#### Axial Coding

During axial coding, short quotes from the literature were systematically grouped into subthemes. Two key conceptual distinctions emerged: (1) themes identifying business model challenges and (2) themes concerning potential strategies to overcome these challenges. Additionally, these themes varied in relevance depending on the stage of business model maturity, whether at the pilot, scaling, or sustaining phase. The expert panel confirmed the practical salience of our challenge vs strategy split and encouraged us to indicate where themes vary by stage of maturity. We kept the research team’s thematic assignments and used reviewer comments to sharpen labels. Panelists did not make inclusion decisions or code data. Their role was advisory, and feedback was incorporated when it improved clarity or semantic completeness. After 3 consecutive coding rounds without new codes or changes to theme relationships, we considered saturation achieved and recorded this in reflexive memos.

#### Selective Coding

In the final (selective coding) phase, concepts developed during axial coding were consolidated into higher-order macroconstructs and clearer distinctions were drawn between (1) internal entrepreneurial challenges, (2) external market challenges, (3) internal business strategies, and (4) external market interventions (Supplemental Tables 6-9, available online at https://www.mcpdigitalhealth.org/).

The emphasis on the necessity for external market interventions including concepts such as strategic partnerships, regulatory frameworks, public procurement, and innovative financing, directed subsequent purposive sampling of new articles and gray literature outside the initial article set. This revealed a significant and strongly correlated subset of mostly gray literature under the umbrella of market-shaping interventions. Consequently, we intentionally incorporated evidence from relevant articles and gray literature pertaining to these 4 types of market-shaping interventions to supplement our findings. Expert panel input at the late stage helped us to verify and connect internal business strategies with external market interventions with no variance among the panel documented. The expert panel and purposive literature selection elaborated subthemes, but no new codes or theme relationships emerged in the final rounds, indicating further conceptual saturation.

## Development of a Synthesizing Argument and Researcher Positionality

Using the CIS process, we identified fundamental business model challenges threatening the sustainability of digital health companies in LMICs, as well as several business strategies and market-shaping interventions for supporting the sustainability of digital health solutions. At key points throughout this process, feedback from the review team, with expertise in digital global health technologies (F.W., M.E., C.P., S.N., B.H., S.H., N.A., and E.I.), international business strategy (B.H., F.W., and E.I.), and complex market-creation (B,H., F.W., and J.H.), was used to ensure multiple perspectives were incorporated into the longitudinal processes of categorization and synthesis.

Although nearly all authorial team has a background in digital health implementation in LMICs, reflexivity remains key during CIS as other individual biases and experiences can affect the interpretive process. These positions can bias researchers to underestimate the difficulties of private sector involvement in LMICs and to overlook cultural, political economy, and historical factors that shape implementation. Thus, the first author (E.I.) kept reflexive memos during coding and synthesis phases to document potential sources of personal bias for reflection with the authorial team and expert panel. Such biases could include the Africa-focused leaning of the authorial team’s expertise, the graduate education received by authors from the global north and the first author’s (E.I.) entrepreneurial background.

Furthermore, we invited feedback from 7 expert panelists with experience of funding or operating digital health companies in LMICs (Supplemental Table 5, available online at https://www.mcpdigitalhealth.org/). The expert panel suggested additional sources and highlighted potential context factors we could have underweighted for us to review back in the literature. The study team retained full control of screening, coding, and interpretation.

Further, we present a conceptual framework depicting the dynamics of our findings on the key challenges and strategies underling the sustainability of digital health markets and business models for global health impact. Short quotations from the extracted literature are used throughout to illustrate key concepts. In the Discussion section, we propose a structured approach to guide stakeholders in shaping digital health markets in LMICs through targeted interventions aligned with their unique public health system goals.

## Results and Conceptual Framework

**Figure 2:**
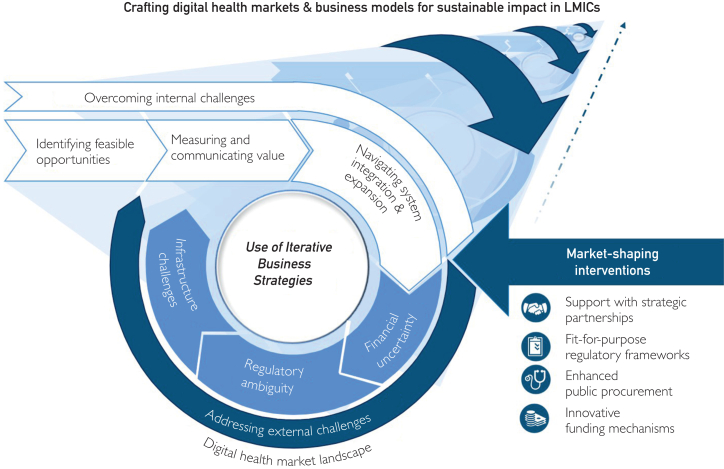
Conceptual framework integrating the challenges of sustaining digital health business models in LMICs with the potential internal strategies for overcoming them and external “market-shaping” interventions for aligning business models with public health impact.

The CIS, including both 21 article extractions (Supplemental Table 1, available online at https://www.mcpdigitalhealth.org/), resulted in the creation of a conceptual framework. This conceptual framework depicts 4 key challenges to the sustainability of digital health business models in LMICs which could potentially be reduced by the strategic deployment of internal business strategies and external market-shaping interventions. The conceptual framework in Figure 2 details these concepts.

Internal threats to sustainability consist of challenges related to managing value creation for complex stakeholder networks, including difficulties related to identifying feasible opportunities, measuring and communicating value, and navigating system integration and expansion. External threats are market challenges including financial uncertainties, infrastructure limitations, and regulatory ambiguity. These overarching challenges encompass additional subchallenges that may vary in severity depending on the technology or country in question. Depending on the context, addressing these challenges may require both iterative business strategies, which differ based off the stage of the company or digital health initiative and external market-shaping interventions in the form of strategic partnerships, fit-for-purpose regulatory frameworks, innovative funding mechanisms, and enhanced public procurement (Supplemental Table 2, available online at https://www.mcpdigitalhealth.org/). Each of the components of the conceptual framework are explored in depth further.

### Key Challenges to the Sustainability of Digital Health Business Models in LMICs

#### Internal Entrepreneurial Challenges

##### Managing Value Creation for Complex Stakeholder Networks

Digital health companies often struggle to identify problem areas that enable both impact and commercial success. Early-stage co-creation of value propositions appealing to diverse stakeholders, patients, providers, donors, implementing partners, and policymakers is constrained by complex incentives and a lack of contextual data to triangulate shared problem areas.^19^^,^^29^^,^^34^ Additionally, differences in resource availability and timelines for impact make prioritizing stakeholder needs into product features particularly challenging.^10^^,^^11^^,^^19^

The evaluation of costs and benefits in e-health and health care in general is a perennial problem involving a large number of different stakeholders and sometimes conflicting views on the value of a particular course of action.^32^

As digital health startups scale, misaligned metrics for success can undermine stakeholder trust, engagement, and financial investment. Variability in data quality, interoperability challenges, and differing outcome measurement standards make it difficult to define economic impact.^32^ Benefits for one stakeholder (eg, patients accessing quicker care via telehealth) may not translate to positive outcomes for another (eg, physicians earning more for physical rather than virtual consultations).^19^^,^^23^^,^^29^

Lastly, even a solution with a growing base of users may have limited benefits at scale if the broader health system lacks the capacity to, for example, manage an increase in patient demand for follow-up care from a virtual triage tool.^5^ Without a more coordinated system of care management (digital or non-digital) to integrate the tool within, many digital health point solutions may only marginally improve the downstream health outcomes they were made to tackle.

#### External Challenges in the Business Environment

##### Financial Uncertainties

Financial uncertainties, especially past pilot stages, continue to stifle the sustainability of digital health solutions in LMICs.^2^^,^^22^^,^^27^^,^^29^^,^^32^ Three key issues exacerbate financial uncertainties: lack of funding, funding unreliability, and high transaction costs. As a former head of e-health for the Ministry of Health in a LMIC, reportedly explained:

Many (mHealth) projects … begin without an idea of who will fund them in the long run. Often, without continued funding from initial donors, high operating costs cause the eventual downfall of the project.^5^

Culturally, the global health financing system, which often prioritizes vertically focused interventions with clear value for money, could have implicit difficulties funding horizontally integrated digital health interventions with less-proven business models or impact timelines.^16^^,^^17^^,^^23^ Furthermore, the context-specific nature of digital health implementation, which often demands longer and more iterative approaches, may conflict with a philanthropic culture of short-term, milestone-driven grant cycles.^5^^,^^17^

High transaction costs, referring to sales, marketing, and payment collection, are another key barrier.^35^ Digital health tools often require frequent training and ongoing support, making it costly to reach and retain users across dispersed populations with infrastructure and language barriers.^5^^,^^23^ Even when digital health companies demonstrate impact, reimbursement inefficiencies can result in delayed payments leading to operational and financial tensions.^17^^,^^23^^,^^31^^,^^35^

#### Infrastructure Challenges

Digital health technologies often require access to cellular or Wi-Fi networks to facilitate consultations or exchange health data. A lack of quality infrastructure, both digital (eg, Wi-Fi, cellular, data exchange, digital payment, and digital ID) and physical (eg, power and devices), can hinder pilot success and scalability.^36^ Chronic infrastructure challenges can degrade service reliability, eroding user trust and retention.

MOTECH, an mHealth system in Ghana, attempted to store health information on employees’ cell phones. However, limited memory capacities forced a significant business model change.^5^

Even when connectivity is available, costs for connectivity are often several magnitudes higher in LMICs compared with other contexts.^5^ This is problematic since many value propositions in digital health require high-volume data exchange as a foundation for quality user engagement.^5^^,^^9^ Furthermore, as companies scale, operational costs for electricity and physical devices can also outpace financial sustainability, especially in LMIC’s where such costs may be too high to pass onto other stakeholders.^5^^,^^9^^,^^10^^,^^27^^,^^33^^,^^34^

In conclusion, digital health companies face tough decisions in managing these infrastructure costs. Whether building in-house systems, relying on third-party providers, or leveraging existing platforms, expenses in LMICs are high, with limited alternatives.^5^^,^^23^^,^^27^ This reality can complicate balancing scalability with financial sustainability.

#### Regulatory Ambiguity

Two key regulatory issues threaten sustainable business models in LMICs: a lack of regulation and regulatory complexities where regulation exists.

The technical nature of DHIs means they are hard for regulators to understand, leading to low capacity for oversight. Absence of country-specific regulatory frameworks often delays market entry and/or or commercialization of products.^10^^,^^31^^,^^34^

HealthKeepers, an mHealth venture in Ghana, faced issues framing themselves as a legal company. People did not want to sign up as employees with HealthKeepers because they did not understand the legal structure of their business model.^5^

In addition, poor harmonization across jurisdictions creates expensive compliance challenges, especially when targeting disparate populations most in need.^23^^,^^34^ Weak coordination among central health authorities often leads to suboptimal regulatory processes, forcing companies managing sensitive data to obtain redundant approvals for data privacy, device certification, or interoperability across geographies.^10^^,^^11^^,^^37^

Lastly, regulations designed for other businesses are often misapplied to digital health companies. For example, laws prohibiting pharmaceutical advertising in India have restricted many telemedicine companies from marketing even nonpharmaceutical services.^36^^,^^37^ Moreover, many telemedicine companies in LMICs also face challenges gaining legal recognition within preexisting regulatory frameworks.^5^

### Strengthening Entrepreneurial Capabilities: Use of Iterative and Fit-for-Purpose Business Strategies

Aimed at entrepreneurs in LMICs, the section below summarizes key strategies for overcoming internal and external business challenges, segmented by where they emerge at different stages of business model maturity. A table summary of these internal entrepreneurial strategies is available in the Supplemental Table 10 (available online at https://www.mcpdigitalhealth.org/). Expert panelists confirmed little variance in the perceived importance of considering such solutions across geographies and technology types.

#### Piloting Business Models in Digital Health

Digital health companies should leverage public reports and interviews with local experts to map key stakeholder interactions and power dynamics in the health system, uncovering pilot opportunities for value creation.^10^^,^^11^^,^^22^^,^^23^ Conducting discovery interviews can also help identify valuable local value propositions and generate insights into why preexisting companies succeeded or failed.^11^^,^^20^^,^^23^^,^^25^

For digital health point solutions, products that target select segments of a health care workflow, extra consideration may be needed to ensure the product integrates well with broader health system factors.^11^^,^^25^ These interconnected factors, such as the capacity of the health system to absorb new patient demand, heavily influence the outcomes these technologies may look to tackle and thus their potential for long-term sustainability.

Once a digital health company has co-created metrics for a pilot project, it will need to ensure that it has the capacity to cost-effectively deliver and evaluate its impact.^10^^,^^11^^,^^25^^,^^27^ Collaborating with academic institutions may help in addressing early gaps in scientific and research expertise.^31^^,^^34^ Meanwhile, where regulation exists, understanding how a product’s intended use will influence its regulatory classification will help ensure the challenges of regulatory compliance are accounted for in the long-term company strategy.^19^^,^^34^ Where regulation is lacking, entrepreneurs should engage regulators with early outcomes data and follow international best practices (eg, WHO Guidelines, GDPR, and HIPAA).^10^^,^^11^^,^^23^

As digital health companies attempt to scale, they must carefully decide which value propositions to develop and when.^11^^,^^19^^,^^31^ To do this cost-effectively, they should use iterative methods like Lean Startup and Agile Development methods.^7^^,^^38^ By continuously testing and refining their offerings using customer and user feedback, companies can ensure they are focused on maximizing user-centric value as cost-efficiently as possible.^7^^,^^9^^,^^38^

Lastly, companies should identify stakeholders with the motivation to become customers and tailor their business models accordingly.^7^ Testing pricing strategies; conducting market research with patients, providers, or partners; and diversifying funding streams can help create financial models to inform strategies for ensuring long-term sustainability.^9^^,^^11^^,^^31^^,^^34^

#### Scaling-Up Business Models in Digital Health

Scaling digital health in LMICs requires continuous monitoring of clinical, operational, and financial metrics to assess impact, optimize cost-effectiveness, and identify new opportunities to deliver greater value at lower costs.^11^^,^^31^^,^^38^ Aligning an interdisciplinary team around these insights will be critical for navigating complex strategic decisions and ensuring long-term success.^10^

As digital health companies expand, rising costs for cellular connectivity, data storage, electricity, and physical devices can quickly become unsustainable.^5^^,^^11^^,^^31^ Store and forward mechanisms, which balance use of local phone storage vs cellular networks based off cellular availability is an example of an enabling frugal approach.^5^ Such mechanisms may be particularly valuable for extending technologies to areas with inconsistent or expensive cellular networks or to workers that have limited memory capacity on their devices.

Beyond technology, empowering local workers and communities can provide a cost-effective way to scale the user base. By leveraging existing social networks, companies can spread awareness, recruit employees, and build community trust.^5^^,^^9^^,^^11^^,^^31^ However, aligning partner and employee incentives is necessary to prevent malpractice, for example, if community health workers began unintentionally overprescribing antibiotics owing to volume-based sales incentives.^5^^,^^9^

Lastly, to ensure financial viability, many digital health companies may introduce ancillary services such as e-pharmacy or laboratory testing to generate additional revenue and cross-subsidize essential but less profitable services, such as maternal health screenings.^9^^,^^11^ Additionally, hybrid pricing models, for example, combining subscription fees with small pay-per-use charges, can help shift revenue streams to different stakeholders depending on their purchasing power.^7^^,^^9^^,^^29^

#### Sustaining Business Models in Digital Health

As companies mature, they should continue to monitor and iteratively act on feedback from users, customers, and partners. Empowering feedback from local employees, partners, and end-users through set processes and incentives makes it easier to add new products or improve existing ones.^11^^,^^28^^,^^31^^,^^38^ As the needs of users evolve over time, strong feedback mechanisms will help anticipate changes, prevent quality control issues, and maintain market share.^7^^,^^11^^,^^38^

Second, well-established companies can consider leveraging their reputation to negotiate lower infrastructure costs. For example, the Apollo Telemedicine Networking Foundation secured free satellite connectivity and hardware through the Indian Space Research Organisation to link teleconsultation services from hospitals to rural health centres in India.^32^

Lastly, by engaging in cooperative competition, private sector coalitions can play a key role in advancing standards for safety, impact measurement, reimbursement, and procurement.^11^^,^^23^ However, public health officials must ensure these efforts do not lead to regulatory capture or new extractive power structures in health care delivery.^11^

### External Market-Shaping Interventions: Development of an Enabling Business Environment

Four types of active market-shaping interventions emerged for fostering a business environment for sustainable digital health business models. These include support with strategic partnerships, fit-for-purpose regulatory frameworks, enhanced public procurement, and innovative financial mechanisms. Table 4 illustrates several potential strategies. A table summary of these external market-shaping interventions is available in the supplementary materials (Supplemental Table 11, available online at https://www.mcpdigitalhealth.org/).

#### Support With Strategic Partnerships

Given the interdisciplinary skill-sets and resources required to deliver digital health solutions, facilitating strategic partnerships can enhance the cost-effectiveness and scalability of digital health initiatives through exchanges of knowledge, capabilities, social capital, and infrastructure.^5^^,^^7^^,^^11^^,^^31^^,^^34^ For instance, externally sponsored initiatives such as incubators, accelerators, and networking events can provide private sector innovators with equitable opportunities to understand the on-the-ground realities of health care delivery.^11^^,^^34^ These platforms can enable innovators to uncover shared pain points in the local health system, build connections across stakeholder networks, and promote the adoption of new products and services.^5^

Moreover, governments can further reduce the costs associated with infrastructure challenges by facilitating cross-industry partnerships with companies in industries such as banking, insurance, and telecommunications.^23^^,^^32^ Such companies already have established digital infrastructure and larger customer bases, which can help improve the scalability of smaller digital health initiatives.^10^ Lastly, fostering proactive partnerships between digital health companies and academic institutions can provide access to the scientific expertise needed to effectively research, monitor, and evaluate the impact of complex digital health interventions.^11^^,^^31^^,^^34^

#### Fit-for-Purpose Regulatory Frameworks

Given the complex fast-moving trajectory of digital health, LMIC governments should prioritize establishing or joining regulatory sandboxes. Regulatory sandboxes are controlled environments that allow policymakers to directly collaborate with the private sector to assess how emerging technologies perform in their local environment and to develop fit-for-purpose regulatory frameworks that balance safety and equity with incentives for local innovation.^7^^,^^11^ This approach may be especially helpful for interdisciplinary technologies like digital health which may require the collaboration of multiple groups of authorities (ie, telecommunications, health services, and labor) to coordinate on interoperability requirements, technical standards, and protocols for patient safety.

In the long-term, regulatory sandboxes could also help enable regulatory harmonization at regional, national or multinational levels.^39^ By aligning digital standards and technical requirements across borders, governments can help simplify the expansion of high-impact digital health technologies, enabling digital health innovations to more cost-effectively scale to new geographies.^6^^,^^7^ Such regulatory efforts should consider the government’s capacity to manage new digital health evaluation and compliance efforts and seek to prevent regulatory capture by commercial interests.

Finally, regulatory mechanisms can also be used to broker access to digital public infrastructure.^6^ The Indian Space Research Organisation has taken this approach by enabling select telemedicine solutions to leverage its satellite infrastructure for serving rural populations.^32^ Such approaches can help dramatically lower the costs of scaling such technologies, especially in LMIC’s with high costs of connectivity.

#### Enhanced Public Procurement

In order to drive public sector demand for digital health technologies with complex implementation timelines, governments could consider carefully selected positive and/or negative financial or nonfinancial incentives.^8^^,^^40^ For example, the Indian National Health Authority launched the Digital Health Incentives Scheme in order to incentivize health facilities, laboratoriess and digital health providers to register patients into their new national electronic health record registry.^41^

However, with an increase in the supply and demand for digital health solutions there also runs the risk of accelerating a more fragmented and inequitable distribution of digital health in the public sector.^6^^,^^42^ To prevent overfragmentation, governments will need to continue to enforce interoperability of health records, services, and claims, while streamlining public sector procurement through efficient but transparent platforms and processes.^6^^,^^7^^,^^43^^,^^44^ To facilitate this, governments should consider focused investments and procurement into digital public infrastructure including infrastructure for digital IDs, digital payments and data exchange.^23^^,^^45^^,^^46^

In addition, governments should consider focusing technology infrastructure investments for digital health adoption in areas of the highest need. Furthermore, governments can consider committing to longer-term contracts or aggregating demand from purchasers across multiple regions, counties, or even countries to negotiate lower costs and expand access to digital health technologies.^6^

Lastly, public entities must establish reliable mechanisms to ensure timely reimbursement of public funds to digital health companies as per contractual agreements. Persistent delays in payment can cripple companies’ ability to pay employees, invest in scaling their solutions, and maintain operations, eroding investors and funder confidence in private sector digital health initiatives in LMICs.^17^

#### Innovative Funding Mechanisms

Although early-stage grants are common in digital health, few mechanisms support companies, especially local founders, through the transition from pilot project to sustainable operations.^34^^,^^47^ To bridge the gap, philanthropists and governments offering nondilutive funding could explore outcomes-based financing models like social and development impact bonds, which tie certain payments to predefined operational or clinical performance metrics.^6^^,^^11^^,^^48^ If designed carefully for third-party adjudication, such guarantees may prove beneficial in aligning private sector investments with sustainable health impact at higher scale.^7^^,^^17^ However, such changes require stronger accountability mechanisms and capacity building for data systems, research, monitoring, and evaluation to ensure outcomes-based reimbursements are valid and emanating from accurate data.^48^

For investors in digital health companies, use of blended and mezzanine funding models can offer more sustainable financing for digital health companies in LMICs. Blended financing combines grants, concessional loans, and equity, often offering flexible repayment terms.^48^ Mezzanine funding, a hybrid of debt and equity financing, provides loans with the option to convert into equity, aligning investor returns with company growth.^47^^,^^48^ Such funding models may help to bridge early-stage funding “valleys of death” in digital health in LMICs by providing more flexible funding for digital health companies, while derisking private sector investments.^47^^,^^48^

## Discussion

Entrepreneurs in LMICs face significant challenges delivering sustainable digital health solutions within complex market ecosystems. To address this, governments and philanthropic organizations could support entrepreneur-level efforts by deploying tailored market-shaping interventions that align private sector success with tangible improvements in public health outcomes. However, implementation of such strategies could vary widely across countries due to factors such as the type of digital health technology prioritized, the availability of technical talent, regulatory capacity, and funding availability within the health care system.

**Figure 3:**
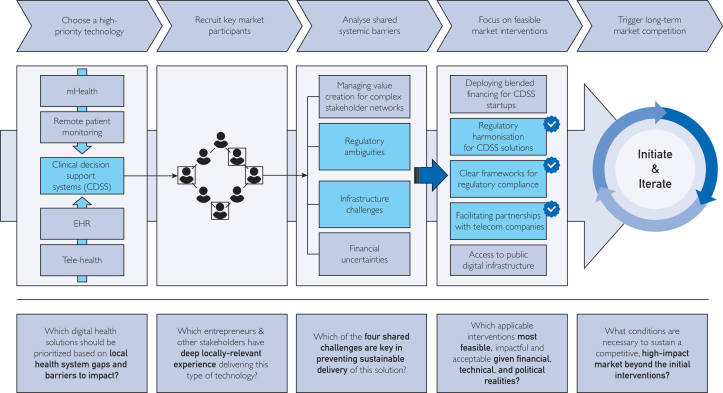
A proposed step-wise digital health market-shaping process to inform market-shaping for digital health for public health by governments and global health funders.

To further inform this process, we have modified an adaptation of the USAID Market-Shaping Pathway previously designed for market-shaping assistive technologies in LMICs by Savage et al.^49^ We apply it to market-shaping digital health technologies in Figure 3 based on our own research into the unique challenges of sustaining digital health business models in LMICs. To make the link between our process and the evidence clear, we use 4 findings from the results as F1–F4: F1 internal challenges managing value creation for complex stakeholder networks, F2 financial uncertainties, F3 infrastructure challenges, and F4 regulatory ambiguity. We state which finding each step addresses.

First, given the diversity of digital health tools and their implementation, it may be important to leverage local experts in order to prioritize a small number of digital health technologies for initial market-shaping activities.^6^ This step addresses F1 by focusing many stakeholders on a small set of clear use cases, and it keeps plans realistic given F2 funding limits, F3 infrastructure gaps, and F4 regulatory requirements. Furthermore, recruiting key market participants, including companies that currently provide or could potentially provide these technologies, will be essential before conducting any diagnostic. Early recruitment supports co-design that reduces F1 misaligned goals and information gaps, and it lowers the effort and risk around payments and contracting linked to F2.

To unearth the key challenges facing the sustainable delivery of these technologies, governments can consider creating regulatory sandboxes to facilitate open dialogue and data exchange between the private and public sector. Sandboxes make F4 rules clear and testable, surface F3 infrastructure limits under real conditions, and help sharpen how value is defined and measured for F1.

Such collaborations may prove crucial in identifying the potential duration, cost, and feasibility of market-shaping activities, allowing regulators and nongovernmental organizations to match interventions that appear both necessary and acceptable to the challenges they have identified from F1 to F4 factors such as the availability of financial resources, technical talent, political will, and stakeholder incentives should be considered when determining which interventions to focus on and when.

Finally, committing to long-term market maintenance through incentives and competition may be important for incentivizing incumbents to continually improve the quality and affordability of their solutions, while also encouraging new market entrants. Continuous initiation and iteration governments and local actors could prove useful in building trust and alignment for F1, keeping payments predictable for F2, supporting shared infrastructure for F3, and preventing regulatory capture while improving regulation for F4.

## Conclusion

Entrepreneurs in LMICs face both internal and external challenges delivering sustainable digital health solutions. Governments and philanthropies could deploy, and subsequently evaluate, tailored market-shaping interventions if they aim to align private sector capabilities toward sustainably improving health systems. Prioritizing technologies, recruiting key participants, analyzing systemic barriers, focusing on feasible interventions, and continuous evaluation, iteration and institutionalization may be crucial in ensuring these markets remain equitable and sustainably focused for public good.

## Potential Competing Interests

The authors report no competing interests.
