# Supplementary material for: Sustainable by Design: Digital Health Business Models for Equitable Global Health Impact in Low-Income and Low-Middle-Income Countries
Source: Mayo Clin Proc Digit Health. 2025 Sep 4;3(4):100261. doi: 10.1016/j.mcpdig.2025.100261 (PMC12547002; doi:10.1016/j.mcpdig.2025.100261)
Supplement: Supplementary Material [file mmc1.docx]

**Supplementary Appendix for “Sustainable by Design: Digital Health Business Models for Equitable Global Health Impact in LMICs”**

**Table of Contents**

1. Table 1: Overview of Extracted Articles
2. Table 2: Summary of the Conceptual Framework
3. Table 3: Search Strings & Databases
4. Table 4: Five Conditions for Quality Appraisal
5. Table 5: Expert Reviewer Background
6. Tables 6-9: Overview of Macro-Theoretical Constructs, Themes, Sub-themes & Examples
7. Table 10: Summary of Internal Entrepreneurial Strategies
8. Table 11: Summary of External Market-Shaping Interventions

**Supplementary Table 1: Overview of Extracted Articles**

| **Key Background Information** | | | | | | **Key Macro-Themes Addressed by the Article** | | | |
| --- | --- | --- | --- | --- | --- | --- | --- | --- | --- |
| **Title** | **Author(s) and Date of Publication** | **Specific DH Technology Focus?** | **Explicitly LMIC focus or General applicability?** | **Study design** | **Business Model Construct/Framework Used or Created** | **(1) Internal Entrepreneurial Challenges** | **(2) External Market Challenges** | **(3) Potential Internal Business Strategies** | **(4) Potential External Market-Shaping Strategies** |
| Business Models for Telemedicine Services: A Literature Review | *Acheampong and Vimarlund, 2015 [31]* | Telehealth | Multiple LMICs | Literature Review | Modified Business Model Canvas | YES | YES | YES | NA |
| Why classic Business Modelling doesn't work for collaborative contexts | *Albert and Van der Auwermeulen, 2017[21]* | No – General DH Technologies | General applicability | Interviews & Workshops | Unique Construct | YES | YES | YES | NA |
| Business model innovation in healthcare: A theoretical perspective | *Babatunde, 2024 [11]* | No – General DH Technologies | General applicability | Systematic Review | Unique Construct | YES | YES | YES | YES |
| Indian Telemedicine Industry: Evolving Nature of Business Models and Customer Interactions | Bhattacharyya et al. 2021 [38] | Telehealth | India | Multiple Case Studies | NA | YES | YES | YES | NA |
| Designing sustainable revenue models for CHW-centric entrepreneurial ventures | *Callan et al., 2014 [9]* | No – General DH Technologies | LMICs | Systematic Review & Expert Opinion | Business Model Canvas | YES | YES | YES | NA |
| Investigating on Requirements for Business Model Representations in the Health IT domain | *Gand et al., 2017 [23]* | No – General DH Technologies | General applicability | Literature Review & Design Approach | Unique Construct | YES | YES | YES | NA |
| Towards conceptual enhancements of the Business Model Canvas for the design of Health IT solutions | *Gand et al., 2018 [22]* | No – General DH Technologies | General applicability | Literature Review & Design Approach | Modified Business Model Canvas | YES | YES | YES | NA |
| Artificial Intelligence as a Growth Engine for Healthcare Startups | *Garbuio and Lin , 2019 [24]* | AI-Technologies | General applicability | Multiple Case Studies & Expert Opinion | Unique Construct | YES | YES | YES | NA |
| Care coordination in a business-to-business and a business-to-consumer telemonitoring case | *Grustam et al., 2017 [37]* | Remote Patient Monitoring | General applicability | Literature Review | Unique Construct | YES | YES | NA | NA |
| Exploring business models for managing uncertainty of new products in medical device and biotechnology firms | *Javanmardi et al., 2024 [33]* | No – General DH Technologies | General applicability | Systematic Literature Review | Modified Business Model Canvas | YES | YES | YES | YES |
| Business approach for IoT based health solutions | *Jog et al., 2015 [30]* | No – General DH Technologies | General applicability | Multiple Case study | Business Model Canvas | YES | NA | YES | YES |
| Business Models for E-Health: Evidence from Ten Case Studies | *Kimble et al., 2015 [34]* | No – General DH Technologies | Mix | Multiple Case Studies | Business Model Canvas | YES | NA | YES | YES |
| Challenges and Success Factors of Business Models in Digital Health: A Narrative Literature Review | *Luur, 2023 [25]* | No – General DH Technologies | General applicability | Narrative Literature Review | Business Model Canvas | YES | YES | YES | YES |
| mHealth Business Model Framework for the Maternal and Baby Segment: A Design Science Research Approach | *Mueller et al., 2019 [26]* | mHealth | General applicability | Design Approach | Unique Construct | YES | YES | NA | NA |
| Business Models in Digital Health: Bibliometric Analysis and Systematic Literature Review | *Pascarelli et al., 2023 [35]* | No – General DH Technologies | General applicability | Systematic Literature Review | Modified Business Model Canvas & Ojala Framework | YES | NA | YES | NA |
| Anatomy of Successful Business Models for Complex Services: Insights from the Telemedicine Field | *Peters et al., 2015 [27]* | Telehealth | General applicability | Design Approach | CompBizMod Framework | YES | YES | YES | NA |
| Developing Sustainable Business Models: A Micro foundational Perspective on the Role of Individuals | *Ringvold et al., 2023 [10]* | Telehealth | Bangladesh | Case Study | NA | YES | YES | YES | YES |
| On the utility of e-health business model design patterns | *Sprenger and Mettler, 2018 [28]* | No – General DH Technologies | General applicability | Design Approach & Focus Groups | Unique Construct | YES | NA | NA | NA |
| Why do entrepreneurial mHealth ventures in the developing world fail to scale? | *Sundin et al., 2016 [5]* | mHealth | LMICs | Literature Review & Multiple Case Studies | NA | YES | YES | YES | YES |
| The 4P telehealth business framework for Iran | *Velayati et al., 2022 [29]* | Telehealth | Iran | Literature Review & Delphi Workshop | Unique Construct (4P Framework) | YES | YES | YES | NA |
| Conceptualizing the landscape of digital health entrepreneurship | *Weimar et al., 2024 [36]* | No – General DH Technologies | General applicability | Systematic Literature Review | Unique Construct | YES | YES | YES | YES |

**Supplementary Table 2: Summary of the Conceptual Framework**

| **Summary of the Conceptual Framework** | | |
| --- | --- | --- |
| ***Internal Entrepreneurial Challenges to Overcome*** | | |
| 1. **Managing Value Creation for Complex Stakeholder Networks** | | |
| **Identifying feasible opportunities for impact:** Challenges identifying opportunities for both sustainable impact and commercial viability. | **Navigating System Integration & Expansion:** Difficulties scaling and making strategic trade-offs when embedding solutions into LMIC health systems. | **Measuring & Communicating Value:** Challenges demonstrating evidence of clinical, operational or economic impact for different stakeholders or customers. |
| ***External Challenges in the Business Environment*** | | |
| **(2) Financial Uncertainties** | **(3) Infrastructure Challenges** | **(4) Regulatory Ambiguity** |
| **Lack of Funding:** Difficulties receiving enough long-term funding to transition from pilot to scale and from scale to sustainability. | **Connectivity Constraints:** Difficulties achieving cost-effective scaling due to limited access to affordable and reliable internet or mobile networks. | **Lack of Regulation:** Navigating regulatory uncertainty in the absence of established legislation. |
| **Funding Unreliability:** Difficulties dealing with rising operational costs with inconsistent funding. | **Device Affordability/Accessibility:** High costs of procuring and maintaining devices necessary to host or support digital health initiatives hinder scalability. | **Regulatory Complexity:** Difficulties complying with complex or fragmented regulatory requirements & processes. |
| **High Transaction Costs:** Difficulties in securing revenue from customers due to administrative complexities, payment delays, or misaligned financial processes. | **Power Reliability Challenges:** Difficulties scaling digital health solutions in regions with inconsistent or limited access to power infrastructure. |  |
| ***Critical Internal & External Strategies to Implement or Maintain*** | | |
| **Internal Strategies:** Improved Entrepreneurial Capabilities | **Use of Iterative and Fit-for-Purpose Business Strategies:** The adoption of entrepreneurial practices that allow for the cost-effective creation of value using iterative methodologies like Lean or Agile. | |
| **External Strategies:** Market-Shaping’ Interventions | **Development or ‘Shaping’ of an Enabling Business Environment:** Creation of a more enabling & subsequently competitive business environment using: Support with Strategic Partnerships, Fit-For-Purpose Regulatory Frameworks, Enhanced Public Procurement and Innovative Financing Mechanisms | |

**Supplementary Table 3: Search Strings & Databases**

5 databases: Full Text Available/English

| **Database (& date of search)** | **Search String** | **Number of articles** |
| --- | --- | --- |
| **Business Source Ultimate**  All Fields  Dates published:  2014-5/14/2024 | (“business model*” OR “business model framework” OR “business model innovation” OR “business strategy*”) AND (“digital health” OR “ehealth” OR “electronic health” OR “e-health” OR “health informatics” OR “medical informatics” OR “mHealth” OR “mobile health” OR “electronic health record” OR “healthcare management system” OR “health financing” OR “health IT” OR “healthcare IT” OR “health information technology” OR “health-tech” OR ”health tech”  OR “tele-health” OR “telemedicine” OR “tele-medicine” OR “digital diagnostics” OR “healthcare AI” OR “healthcare artificial intelligence” OR “Software as a Medical Device” OR “SaMD”) | 112 |
| **Pubmed**  All fields  Dates published:  2014-5/14/2024 | Search((“business model*” OR “business model framework” OR “business model innovation” OR “business strategy*”)) AND (“digital health” OR “ehealth” OR “electronic health” OR “e-health” OR “health informatics” OR “medical informatics” OR “mHealth” OR “mobile health” OR “electronic health record” OR “healthcare management system” OR “health financing” OR “health IT” OR “healthcare IT” OR “health information technology” OR “health-tech” OR ”health tech”  OR “tele-health” OR “telemedicine” OR “tele-medicine” OR “digital diagnostics” OR “healthcare AI” OR “healthcare artificial intelligence” OR “Software as a Medical Device” OR “SaMD”) Sort by: Best Match | 162 |
| **Web of Science**  Topic search  NOT Database:Preprint Citation Index  Dates published:  2014-5/14/2024 | TOPIC: (“business model*” OR “business model framework” OR “business model innovation” OR “business strategy*”) AND TOPIC: (“digital health” OR “ehealth” OR “electronic health” OR “e-health” OR “health informatics” OR “medical informatics” OR “mHealth” OR “mobile health” OR “electronic health record” OR “healthcare management system” OR “health financing” OR “health IT” OR “healthcare IT” OR “health information technology” OR “health-tech” OR ”health tech”  OR “tele-health” OR “telemedicine” OR “tele-medicine” OR “digital diagnostics” OR “healthcare AI” OR “healthcare artificial intelligence” OR “Software as a Medical Device” OR “SaMD”) | 408 |
| **SCOPUS**  Title/Abs/Key  Dates published:  2014-5/14/2024 | (TITLE-ABS-KEY (“business model*” OR “business model framework” OR “business model innovation” OR “business strategy*”) AND  TITLE-ABS-KEY (“digital health” OR “ehealth” OR “electronic health” OR “e-health” OR “health informatics” OR “medical informatics” OR “mHealth” OR “mobile health” OR “electronic health record” OR “healthcare management system” OR “health financing” OR “health IT” OR “healthcare IT” OR “health information technology” OR “health-tech” OR ”health tech”  OR “tele-health” OR “telemedicine” OR “tele-medicine” OR “digital diagnostics” OR “healthcare AI” OR “healthcare artificial intelligence” OR “Software as a Medical Device” OR “SaMD”)) | 472 |
| **Google Scholar**  Dates published:  2014-5/14/2024 | ("business model”) AND (“digital health”) AND (“global health”) | First 200 |

**Supplementary Table 4: Five Conditions for Quality Appraisal**

| **Appraisal prompts for informing judgements about quality of papers (Dixon-Woods et al. 2006)** |
| --- |
| 1. Are the aims and objectives of the research clearly stated? |
| 1. Is the research design clearly specified and appropriate for the aims and objectives of the research? |
| 1. Do the researchers provide a clear account of the process by which their findings were reproduced? |
| 1. Do the researchers display enough data to support their interpretations and conclusions? |
| 1. Is the method of analysis appropriate and adequately explicated? |

**Supplementary Table 5:** Expert Reviewer Background

| **Initials** | **Professional Role** | **Description of Primary Organisation(s)** | **Geography of Focus** |
| --- | --- | --- | --- |
| MV | *Chief Executive Officer (Startup)* | Large Telemedicine Startup | A LMIC in Southeast Asia |
| SL | *Chief Executive Officer (Startup)* | Small/Medium-Sized HealthIT Startup | A LMIC in Sub-Saharan Africa |
| HN | *Chief Strategy Officer (Startup)* | Large Telemedicine Startup | A LMIC in Southeast Asia |
| CM | *Chief Executive Officer (Funder & Implementing Partner)* | Large Development-Focused Startup Incubator | LMICs World-Wide |
| MS | *Chief Executive Officer (Funder & Implementing Partner)* | Global Health NGO Focused on Market-Shaping | LMICs World-Wide |
| ST | *Chief Executive Officer (Investor)* | Healthcare/Digital Health Venture Capital Firm | Sub-Saharan Africa At-Large |
| PA | *PhD Candidate & Digital Health Consultant (10 yrs)* | Multiple Large Conglomerates & Healthcare Firms | A LMIC in South Asia |

**Supplementary Tables 6-9:** Overview of Macro-Theoretical Constructs, Themes, Sub-themes & Examples

| **Macro-Construct #1: Understanding Internal Entrepreneurial Challenges** | | | |
| --- | --- | --- | --- |
| ***Core Theme*** | ***Subthemes*** | ***Example Articles*** | ***Example Quote*** |
| ***Managing Value Creation for Complex Stakeholder Networks*** | **Challenges Identifying Feasible Opportunities** | *Albert and Van der Auwermeulen, 2017; Garbuio and Lin, 2019; Babatunde, 2024;* | Garbuio and Lin, 2019 -  ***"For a new business, we recommend looking at low-hanging fruit, such as improvements in operational effectiveness and solving inefficiencies. It is exciting to solve big problems, such as optimizing patient treatments, but they also carry important regulatory hurdles, difficulty in acquiring patient data, and data cleaning."*** |
|  | **Challenges Measuring & Communicating Value** | *Acheampong and Vimarlund, 2015; Kimble, 2015; Luur, 2023* | Kimble, 2015 - ***"The evaluation of costs and benefits in e-heath and healthcare in general is a perennial problem involving a large number of different stakeholders and sometimes conflicting views on the value of a particular course of action."*** |
|  | **Challenges Navigating System Integration & Expansion** | *Sundin et al.,2016; Gand, 2018; Babatunde, 2024;* | Babatunde, 2024 -  ***"Integrating innovative healthcare solutions with existing healthcare systems, workflows, and technologies can be challenging due to interoperability issues, data silos, and legacy infrastructure."*** |
| **Macro-Construct #2: Understanding External Market Challenges** | | | |
| ***Core Themes*** | ***Subthemes*** | ***Example Articles*** | ***Example Quote*** |
| ***Financial Uncertainty*** | **Lack of Funding** | *Sundin et al.,2016; Velayati et al., 2022; Luur, 2023;* | Velayati et al., 2022 -  ***"For example, in Iran, the commercialization of telehealth technology has been hindered by several factors such as physician resistance and insufficient funding to develop and support telehealth systems."*** |
|  | **Funding Unreliability** | *Sundin et al.,2016; Luur et al. 2023; Weimar et al., 2024;* | Sundin et al., 2016 -  ***"Often, without the continued funding from initial donors, high operating costs cause the eventual downfall of the [mHealth] project."*** |
|  | **High Transaction Costs** | *Grustam et al., 2017; Luur, 2023; Javanmardi et al., 2024;* | Luur, 2023 -  ***"There are also delays and desynchronization of funding flows in public sector procurement."*** |
|  |  |  |  |
| ***Infrastructure Challenges*** | **Connectivity Constraints** | *Callan et al., 2014; Sundin et al. ,2016; Bhattacharyya et al., 2021;* | Sundin et al., 2016 -  ***"With poor cell phone infrastructure, cell carriers charge extra for roaming and these costs can quickly add up with a high volume of text messaging."*** |
|  | **Device Affordability/Accessibility** | *Callan et al., 2014; Sundin et al. ,2016; Bhattacharyya et al., 2021;* | Callan et al., 2014 -  ***"As organizations move to monetize services, the feasibility of device acquisition and maintenance becomes a pressing concern."*** |
|  | **Power Availability/Reliability** | *Callan et al., 2014; Sundin et al. ,2016; Velayati et al., 2022;* | Sundin et al., 2016 -  ***Living Goods, an mHealth venture based in Uganda, "found that the inability of both clients and employees to charge their cell phones limited their customer base."*** |
|  |  |  |  |
| ***Regulatory Ambiguity*** | **Lack of Regulation** | *Velayati et al., 2022; Luur, 2023; Weimar et al., 2024;* | Weimar et al., 2024 -  ***"Although several contributions exist, regulatory paths are often unclear or have not been defined yet (Rassi-Cruz et al. 2022), and more research is needed to understand how startups can cope with the challenges."*** |
|  | **Regulatory Complexity** | *Bhattacharyya et al. 2021; Luur, 2023; Weimar et al., 2024;* | Bhattacharyya et al., 2021 -  ***"Prior to the COVID-19 pandemic, the GOI had not published clear guidelines for services like teleconsultations and telemedicine ...The norms and protocols related to physician–patient relationship, issues of liability and negligence, evaluation, management and treatment, informed consent, medical records, privacy and security of patient records were ambiguous."*** |
|  |  |  |  |
| **Macro-Construct #3: Potential Internal Business Strategies** | | | |
| ***Core Themes*** | ***Subthemes*** | ***Example Articles*** | ***Example Quote*** |
| **Value Propositions** | **Map stakeholder interactions and power dynamics. (Pilot stage)** | Albert and Van der Auwermeulen, 2017; Javanmardi et al., 2024; Weimar et al., 2024; | Albert and Van der Auwermeulen, 2017 - ***"Clarity regarding how these stakeholders make decisions and how potential incentives for them would look like was prioritized by all encountered companies."*** |
|  | **Conduct discovery work/interviews to identify and co-create valuable solutions. (Pilot stage)** | Albert and Van der Auwermeulen, 2017; Babatunde, 2024; Weimar et al., 2024; | Weimar et al., 2024 -  ***"Research has shown that it is essential for health startups to engage with stakeholders early in the development process (Barlow et al. 2006; Nilsen et al. 2020), as failing to do so can lead to conflicting perspectives and interests (Lyles et al. 2021)."*** |
|  | **Ensure integration with broader healthcare processes and incentives. (Pilot stage)** | Peters et al., 2015; Albert and Van der Auwermeulen, 2017; Gand, 2017; | Gand, 2017 -  ***"The HIT implementation has to fit in the existing care structures and possibly result in innovative care modes. Hence, the development of HIT solutions goes beyond solely technical design. It also has to include strategic aspects to ensure solutions that are embedded in the respective care setting."*** |
|  |  |  |  |
|  | **Continuously test and refine value propositions based off feedback. (Scaling Up)** | Ringvold, 2023; Babatunde, 2024; Weimar et al., 2024; | Ringvold et al. 2023 -  ***"Testing of ideas are an important part of the “searching for SBM [Sustainable Business Models] solutions” phase. These processes contribute to in-depth problem understanding to further develop the product or service and to be able to deliver the service in a manner that brings value to the customer."*** |
|  |  |  |  |
|  | **Use feedback mechanisms to anticipate changes and prevent quality control issues. (Sustaining)** | Luur, 2023; Ringvold, 2023; Babatunde, 2024; | Ringvold et al. 2023 -  ***"Soliciting feedback from patients and caregivers, and using it to drive continuous improvement and innovation, is essential for delivering patient-centered care. Entrepreneurs can leverage patient feedback mechanisms, such as surveys, focus groups, and patient advisory councils, to identify areas for improvement, prioritize initiatives, and measure outcomes."*** |
|  |  |  |  |
| **Value Creation** | **Co-create metrics and expected outcomes with funders, users & customers. (Pilot stage)** | Callan et al., 2014; Babatunde, 2024; Weimar et al., 2024; | Callan et al., 2014 -  **"*When money is tied to a particular outcome, there should be validation that the venture’s products will not only be financially sustainable but will also make a positive social impact as well. If a venture can effectively demonstrate the feasibility and social impact of their products, then acquiring start-up capital should be easier. The measurement of such metrics should be an integral part of the operations of the venture."*** |
|  | **Incorporate regulatory compliance into timelines and trade-offs for product development. (Pilot stage)** | Luur, 2023; Javanmardi et al. 2024; Weimar et al., 2024; | Javanmardi et al. 2024 -  ***"The important issue is that the fields of healthcare and medical equipment require a very long time for technology development, clinical tests, issuance of permits, insurance registration, and distribution to be finally commercialized, and because of this situation they need a sustainable [product] strategy."*** |
|  | **Conduct robust research, monitoring and evaluations. (Pilot stage)** | Luur, 2023; Babatunde, 2024; Weimar et al., 2024; | Weimar et al., 2024 -  ***"It may be advisable for healthcare startups to invest in clinical validation efforts to increase trust in their products, as relying solely on internal data may not be sufficient to establish their validity (Cristea et al. 2019)."*** |
|  | **Use iterative business methodologies like Lean Startup & Agile Development. (Pilot stage)** | Eppley et al., 2021; Ringvold, 2023; Weimar et al., 2024 | Weimar et al., 2024 -  ***"Recently, there has been a shift towards methods that guide entrepreneurs in designing digital health solutions. This marks the transition from classical product development as conducted in hardware-heavy medical technology companies (waterfall method) towards agile methods needed to develop digital solutions."*** |
|  |  |  |  |
|  | **Regularly align an interdisciplinary team around key performance indicators. (Scaling Up)** | Velayati et al., 2022; Ringvold, 2023; Babatunde, 2024; | Babatunde, 2024 -  ***"Forecasting revenue projections and monitoring key performance indicators, such as customer acquisition costs, lifetime value, and churn rates, is essential for revenue model optimization (Gupta et al., 2006). Entrepreneurs should continuously evaluate and refine their revenue model based on market feedback, competitive dynamics, and business performance to maximize revenue growth and profitability over time"*** |
|  | **Implement frugal technical approaches to enable cost-effective scale. (Scaling Up)** | Sundin et al. ,2016; Gand, 2017; Javanmardi et al., 2024; | Javanmardi et al., 2024 -  ***"More specifically, to successfully compete in the segments of emerging markets, companies must come up with resource-constrained innovations and BMs [Business Models] that can create a high value against low costs."*** |
|  |  |  |  |
|  | **Leverage brand reputation to lower infrastructure and other costs through partnerships. (Sustaining)** | Sundin et al. ,2016; Luur, 2023; Babatunde, 2024; | Sundin et al., 2016 -  ***"The health venture Securing Ugandans Right to Essential Medicines (SURE) teamed with Makerere University to conduct their MMS (Multimedia Messaging Service) training workshops. With this partnership, SURE was able to train 113 people in one year, a feat that was only achievable by partnering with Makerere University."*** |
|  | **Engage in cooperative competition. (Sustaining)** | Sundin et al. ,2016; Babatunde, 2024; Javanmardi et al., 2024; | Javanmardi et al., 2014 -  ***"In open innovation BMs... companies active in one industry, those active in several completely different industries, and even competitors may have cooperation with each other. Collaborating companies in such BMs contribute to the development of research and innovation projects, by collectively providing the resources."*** |
|  |  |  |  |
| **Value Communication & Delivery** | **Establish partnerships to cost-effectively reach an initial group of users. (Pilot stage)** | Callan et al., 2014; Ringvold et al., 2023; Luur, 2023; | Ringvold et al. 2023 -  ***"Telenor [ Digital Health Company] used Grameenphone’s [Tele-Com Company] go-to market structure to drive the new service’s distribution and reach. Grameenphone customers who qualified as revenue customers and enrolled in the new service received a basic bundle of health services for free."*** |
|  | **Engage regulators in co-creation and data-sharing with regulators. (Pilot stage)** | Luur, 2023; Ringvold et al. 2023; World Bank (Regulatory Sandboxes for Digital Health); | World Bank (Regulatory Sandboxes for Digital Health) - ***"Innovators and entrepreneurs may also contribute to the development of new regulations and to the revision of existing regulations through participation in sandboxes."*** |
|  |  |  |  |
|  | **Further empower local partners, users and other stakeholders in promoting the product or service. (Scaling Up)** | Callan et al., 2014; Sundin et al., 2016; Ringvold et al., 2023; | Sundin et al., 2016 -  ***"By establishing a connection between customers and the surrounding communities, telemedicine systems can better market themselves as both a health service and as a community resource that people can leverage for a variety of different needs."*** |
|  |  |  |  |
|  | **Advocate for more effective public-sector procurement/collaboration. (Sustaining)** | Velayati et al., 2022; Luur, 2023; Javanmardi et al., 2024; | Javanmardi et al., 2014 -  ***"Companies in the medical device, biotechnology, and healthcare sectors may draw on governmental subsidiaries or financial aids, tax advantages, science and technology parks, and governmental research grants. Public or governmental budgets for R&D can reduce private costs and potentially turn non-profitable projects into profitable ones."*** |
|  |  |  |  |
| **Value Capture** | **Identify stakeholders with motivation to become customers. (Pilot stage)** | Albert and Van der Auwermeulen, 2017; Babatunde, 2024; Javanmardi et al., 2024; | Babatunde, 2024 -  ***"Understanding the needs, preferences, and pain points of target customers is essential for designing a compelling value proposition."*** |
|  | **Test pricing strategies and conduct market research. (Pilot stage)** | Callan et al., 2014; Jog et al., 2015; Sundin et al., 2016;; | Sundin et al., 2016 -  ***"The price points must be determined by pilot tests with paying customers rather than relying on conducting surveys or assuming appropriate costs. CycleTel, a telemedicine system based in India, initially offered its services for free. After market tests with customers, the venture eventually found that INR 30 (USD 0.50) was an appropriate price to charge for the service."*** |
|  | **Consider providing ancillary services to cross-subsidize essential but less profitable services. (Scaling Up)** | Acheampong and Vimarlund, 2015; Jog et al., 2015; Bhattacharyya et al., 2021; | Bhattacharyya et al., 2021 - ***"The telemedicine firms also operate e-pharmacies and provide options for booking diagnostic tests to earn additional revenue."*** |
|  | **Offer hybrid pricing models to maximize revenue streams and accommodate for variation in customer purchasing power. (Scaling Up)** | "Acheampong and Vimarlund, 2015; Jog et al., 2015; Bhattacharyya et al., 2021;" | Bhattacharyya et al., 2021 -  ***"It was found by the authors that telemedicine firms in India generally used hybrid business models, providing options of pay-per-use model, subscription and freemium models."*** |
|  | **Advocate for more effective reimbursement or financing/funding mechanisms. (Scaling Up)** | Velayati et al., 2022; Luur, 2023; Javanmardi et al., 2024;" | Luur, 2023 -  ***"Financing is a key challenge for digital healthcare platforms. Since these platforms often operate driven by a 'social purpose,' their scaling can be slower and less attractive to private [versus public] investors"*** |
|  |  |  |  |
| **Macro-Construct #4: Potential Market-Shaping Strategies** | | | |
| ***Core Themes*** | ***Subthemes*** | ***Example Articles*** | ***Example Quote*** |
| **Support with Strategic Partnerships** | **Facilitate Knowledge Exchange via Incubators, accelerators etc.** | Babatunde, 2024; Weimar et al., 2024; McKinsey & Company (How Digital Tools could boost efficiency in African health Systems); | Weimar et al., 2024 -  ***"Njoku et al. (2023) found that accelerator programmes play a significant role in supporting the adoption of digital health technologies by small- and medium-sized enterprises."*** |
|  | **Broker Academic Partnerships** | Weimar et al., 2024; McKinsey & Company (How Digital Tools could boost efficiency in African health Systems); World Bank (Digital-in-Health Unlocking the Value for Everyone) ; | Weimar et al., 2024 -  ***"Alliances between startups and other organizations, particularly academia-industry alliances, contribute to the success of digital health products (Kikuchi et al. 2021; Capponi and Corrocher 2022) and can even be a basis for receiving grants or support (Ford et al. 2019, 2021)."*** |
|  | **Broker Cross-Industry Partnerships (ie. telecom/banking)** | Sundin et al. ,2016; Javanmardi et al., 2024; World Bank (Digital-in-Health Unlocking the Value for Everyone) ; | World Bank (Digital-in-Health Unlocking the Value for Everyone) -  ***"Productive partnerships will be essential to success. This includes new types of working arrangements and partnerships with and among private sector partners, as well as with stakeholders supporting wider digital transformation efforts outside the health sector."*** |
|  |  |  |  |
| **Fit-for-Purpose Regulatory Frameworks** | **Organise Regulatory Sandboxes** | Babatunde, 2024; World Bank (Regulatory Sandboxes for Digital Health); World Bank (Digital-in-Health Unlocking the Value for Everyone) ; | Babatunde, 2024 -  ***"Policymakers can establish regulatory sandboxes or innovation hubs to provide a safe and flexible environment for testing and scaling innovative healthcare solutions (Omaghomi et al., 2024). Regulatory sandboxes allow startups to experiment with new business models, technologies, and care delivery models under regulatory supervision, fostering innovation while ensuring patient safety and quality of care."*** |
|  | **Create Fit-for-Purpose Technical & Non-technical Standards** | Babatunde, 2024; McKinsey & Company (Building inclusive digitally-enabled health systems in LMICs); Arthur D. Little (Catalyzing digital health India 2024); | Babatunde, 2024 -  ***"Establishing interoperability standards, data exchange protocols, and privacy frameworks can facilitate seamless integration of digital health solutions, improve care coordination, and enhance patient outcomes while protecting patient privacy and security (Ogugua et al., 2024)."*** |
|  | **Engage in Regional Regulatory Harmonization** | World Bank (Digital-in-Health Unlocking the Value for Everyone) ; Digital Square (Digital Public Infrastructure for Health); McKinsey & Company (Building inclusive digitally-enabled health systems in LMICs); | World Bank (Digital-in-Health Unlocking the Value for Everyone) -  ***"Regulatory improvements at national and international levels that enable personal data transfers across borders with data protection and security safeguards are critical to harnessing the potential of crossborder digital health innovations."*** |
|  | **Share Public Technology Infrastructures (e.g., satellite networks)** | Digital Square (Digital Public Infrastructure for Health); World Bank (Digital-in-Health Unlocking the Value for Everyone) ; McKinsey & Company (Building inclusive digitally-enabled health systems in LMICs); | McKinsey & Company (Building inclusive digitally-enabled health systems in LMICs) - ***"Governments and other stakeholders can capitalize on the rapid growth of mobile and internet penetration in LMICs by leveraging existing digital infrastructure, including mobile networks, digital payment systems, and digital identity solutions."*** |
|  |  |  |  |
| **Enhanced Public Procurement** | **Drive Efficiencies in Contracting & Public Procurement** | World Bank (Digital-in-Health Unlocking the Value for Everyone) ; Digital Square (Digital Public Infrastructure for Health); McKinsey & Company (Building inclusive digitally-enabled health systems in LMICs); | World Bank (Digital-in-Health Unlocking the Value for Everyone) -  ***"Traditional procurement can be limited by short timeframes and a lack of focus on outcome measurement and benefit realization, which are crucial for digital health initiatives. One-year contracts, for example, do not align with the rollout of digital health solutions."*** |
|  | **Create Incentives for Public-Sector Digital Health Adoption** | Luur, 2023; Arthur D. Little (Catalyzing digital health India 2024);  World Bank (Digital-in-Health Unlocking the Value for Everyone); | Arthur D. Little (Catalyzing digital health India 2024) -  ***"India’s government has designed and implemented several initiatives to push digitalization through ABDM [Ayushman Bharat Digital Mission]. Simultaneously, the Digital Health Incentives Scheme (DHIS) aims to reward users of ABDM through financial incentives for crossing certain established thresholds of health record linking with ABHA."*** |
|  | **Deploy Investments in Technology Infrastructure** | "Digital Square (Digital Public Infrastructure for Health); World Bank (Digital-in-Health Unlocking the Value for Everyone); McKinsey & Company (How Digital Tools could boost efficiency in African health Systems);" | "Digital Square (Digital Public Infrastructure for Health) -  ***"Connectivity, access to mobile network infrastructure for basic mobile messaging capability, and, for analytics use cases, cloud computing infrastructure similarly will need to be part of national DPI to support DPI-H at scale."*** |
|  |  |  |  |
| **Innovative Funding Mechanisms** | **Consider Outcomes or Performance-based Payment/Funding Models** | Babatunde, 2024; World Bank (Digital-in-Health Unlocking the Value for Everyone); Sattva Knowledge Institute (Digital Health Financing for Health Equity); | Babatunde, 2024 -  ***"Policymakers should consider reforms to reimbursement models and payment mechanisms to incentivize value-based care, preventive interventions, and innovative healthcare solutions (Ehimuan et al., 2024). Aligning reimbursement with quality outcomes, patient satisfaction, and cost containment can encourage healthcare organizations to invest in innovation and adopt more efficient and effective care delivery models."*** |
|  | **Utilise or Test Innovative Financing (e.g. Blended, Mezzanine instruments) by Digital Health Investors** | Sattva Knowledge Institute (Digital Health Financing to Accelerate Health Equity) Stanford BioDesign (HealthTech in East Africa: An Ecosystem Overview); Rethink Priorities Ltd (An Overview of Market-Shaping in Global Heath); | Sattva Knowledge Institute (Digital Health Financing for Health Equity) -  ***"On the supply side, different financing instruments can make digital health solutions available to the underserved by providing upfront capital, supporting providers with risk capital during the trial phase, and returnable grants or result-based financing to demonstrate the value of such solutions. Low-cost or concessional debts can enable the digitisation of low-cost private healthcare facilities, while citizen demand can be accelerated through social or development impact bonds."*** |

**Supplementary Table 10: Summary of Internal Entrepreneurial Strategies**

| **Summary of Key Entrepreneurial Strategies for Digital Health Companies in LMICs** | | | |
| --- | --- | --- | --- |
| **Stages of Startup Maturity** | | | |
| **Business Model Component** | ***Pilot-stage*** | ***Scaling-up*** | ***Sustaining*** |
| *Value Proposition* | - **Map stakeholder interactions** and power dynamics. - **Conduct discovery interviews** to identify and co-create valuable solutions. - **Ensure integration** with broader healthcare processes and incentives. | - **Continuously test and refine value propositions** based off feedback. | - **Use feedback mechanisms to anticipate changes** and prevent quality control issues. |
| *Value Creation* | - **Co-create metrics** and expected outcomes with funders, users & customers. - **Incorporate regulatory compliance** into timelines and trade-offs for product development. - **Use iterative business methodologies** like Lean Startup & Agile Development. - **Conduct robust research**, monitoring and evaluations. | - **Regularly align an interdisciplinary team around key performance indicators** (i.e. clinical, operational and financial metrics) to drive strategic decision-making. - **Implement frugal technical approaches to enable cost-effective scale** (e.g., Store and Forward mechanisms). | - Leverage brand reputation to **lower infrastructure and other costs through partnerships.** - Engage in **cooperative competition** to institutionalise new industry standards. |
| *Value Communication & Delivery* | - **Establish partnerships** to cost-effectively reach an initial group of users. - **Engage regulators** with early outcomes data where regulation is lacking. | - **Further empower local partners, users and other stakeholders** in promoting the product or service. | - **Advocate for more effective public-sector procurement.** |
| *Value Capture* | - **Identify stakeholders with motivation to become customers.** - **Test pricing strategies** and conduct market research. | - **Consider providing ancillary services** to cross-subsidize essential but less profitable services. - **Offer hybrid pricing models to maximize revenue streams** and accommodate for variation in customer purchasing power. | - **Advocate for more effective reimbursement mechanisms** |

**Supplementary Table 11: Summary of Market-Shaping Interventions**

| **Summary of Market-Shaping Strategies for Enabling Digital Health Markets in LMICs** | | | | |
| --- | --- | --- | --- | --- |
|  | ***Support with Strategic Partnerships*** | ***Fit-for-Purpose Regulatory Frameworks*** | ***Enhanced Public Procurement*** | ***Innovative Funding Mechanisms*** |
| ***Addressing Internal Challenges*** |  |  |  |  |
| *Managing Value Creation for Complex Stakeholder Networks* | Facilitate **stakeholder knowledge exchange and alignment** through incubators, accelerators, and networking events.  Engage **academic institutions** to provide scientific expertise and evaluation capacity. | Establish **regulatory sandboxes** to collaboratively test innovations under relaxed requirements. | **Articulate key gaps and priorities** in the public health system  Use of **transparent Procurement** Platforms and Processes  Use **positive and negative incentives** to encourage digital health adoption in the public-sector. |  |
| ***Addressing External Challenges*** |  |  |  |  |
| *Regulatory Ambiguity* |  | Co-develop **locally relevant regulatory frameworks**.  **Clearly articulate and enforce technical standards** for interoperability, privacy and data ownership.  Enable **regional regulatory harmonisation** to support cross-border scaling. |  |  |
| *Financial Uncertainties* |  |  | **Aggregate demand across regions** to negotiate lower costs and expand access.  Commit to **longer-term contracts** to account for implementation challenges.  **Disburse timely payments** for contracted products and services. | Introduce **outcomes or performance-based financing** linked to operational or clinical metrics.  Deploy **blended and mezzanine financing models** to help bridge funding gaps. |
| *Infrastructure Challenges* | Build **cross-industry partnerships** (e.g., telecommunications, banking) to share infrastructure and expand reach. | Subsidise access **to public technology infrastructures** (e.g., satellite networks) for underserved populations. | **Focused investments in digital public infrastructure** (e.g., data ID, digital payment, data exchange).  **Focused investments in technology infrastructure** for the most underserved health system users |  |
